# Preparing Public Health Professionals to Make Evidence-Based Decisions: A Comparison of Training Delivery Methods in the United States

**DOI:** 10.3389/fpubh.2018.00257

**Published:** 2018-09-13

**Authors:** Rebekah R. Jacob, Kathleen Duggan, Peg Allen, Paul C. Erwin, Kristelle Aisaka, Samuel C. Yang, Ross C. Brownson

**Affiliations:** ^1^Prevention Research Center in St. Louis, Brown School, Washington University in St. Louis, St. Louis, MO, United States; ^2^Focus Pointe Global, St. Louis, MO, United States; ^3^School of Public Health, The University of Alabama at Birmingham, Birmingham, AL, United States; ^4^JED Foundation, New York, NY, United States; ^5^Division of Public Health Sciences, Department of Surgery and Alvin J. Siteman Cancer Center, Washington University School of Medicine, Washington University in St. Louis, St. Louis, MO, United States

**Keywords:** evidence-based decision making, public health workforce training, training approaches, evidence-based practice, public health department

## Abstract

**Background:** Evidence-based decision making (EBDM) in health programs and policies can reduce population disease burden. Training in EBDM for the public health workforce is necessary to continue capacity building efforts. While in-person training for EBDM is established and effective, gaps in skills for practicing EBDM remain. Distance and blended learning (a combination of distance and in-person) have the potential to increase reach and reduce costs for training in EBDM. However, evaluations to-date have focused primarily on in-person training. Here we examine effectiveness of in-person trainings compared to distance and blended learning.

**Methods:** A quasi-experimental pre-post design was used to compare gaps in skills for EBDM among public health practitioners who received in-person training, distance and blended learning, and controls. Nine training sites agreed to replicate a course in EBDM with public health professionals in their state or region. Courses were conducted either in-person (*n* = 6) or via distance or blended learning (*n* = 3). All training participants, along with controls, were asked to complete a survey before the training and 6 months post-training. Paired surveys were used in linear mixed models to compare effectiveness of training compared to controls.

**Results:** Response rates for pre and post-surveys were 63.9 and 48.8% for controls and 81.6 and 62.0% for training groups. Participants who completed both pre and post-surveys (*n* = 272; 84 in-person, 67 distance or blended, and 121 controls) were mostly female (89.0%) and about two-thirds (65.3%) were from local health departments. In comparison to controls, overall gaps in skills for EBDM were reduced for participants of both in-person training (β = −0.55, SE = 0.27, *p* = 0.041) and distance or blended training (β = −0.64, SE = 0.29, *p* = 0.026).

**Conclusions:** This study highlights the importance of using diverse methods of learning (including distance or blended in-person approaches) for scaling up capacity building in EBDM. Further exploration into effective implementation strategies for EBDM trainings specific to course delivery type and understanding delivery preferences are important next steps.

## Introduction

The US public health system is complex and includes several key organizations with diverse functions. Governmental health departments (state, local, tribal and territorial) hold primary responsibility for health under the US and state constitutions and directly or indirectly provide disease prevention services to communities such as health screenings, health education, and conduct surveillance. The 51 state health departments (1 per state and the District of Columbia) along with nearly 3,000 local health departments are diverse in the populations they serve (rural, urban, etc.) and in funding and resources available to them (number of full-time employees, partner organizations, etc.) (1, 2). Health departments play a key role in determining programs and policies to keep local communities or larger populations healthy.

Making programmatic and policy decisions based upon the best available research evidence, or evidence-based decision making (EBDM) (3–6), can further the ability to decrease the burden of disease in populations. The systematic application of principles of EBDM and training and capacity building for EBDM vary widely across the globe (7). Many of the fundamental tenets of EBDM originated in Australia, Canada, and the US. Among four countries recently surveyed regarding knowledge and use of EBDM principles, knowledge was highest in the US, followed closely by Australia and Brazil, with much lower knowledge in China (8). Consensus among the public health field exists regarding EBDM as a Core Competency needed among the US workforce (9). The US Public Health Accreditation Board's Standards and Measures also emphasize the importance of having a skilled workforce by requiring accreditation-seeking health departments to demonstrate that they “identify and use the best available evidence for making informed public health practice decisions” (standard 10.1) and “promote understanding and use of the current body of research results, evaluations, and evidence-based practices with appropriate audiences” (standard 10.2) (10). Even so, gaps in skills for EBDM remain (11). The spread of EBDM is crucial among the public health workforce charged with planning, implementing, and evaluating programs and policies for disease prevention. However, the EBDM process, particularly within the context of US governmental public health agencies, is met with several barriers. Individual-level barriers include skills needed to carry out EBDM, e.g., prioritizing programs or policies, and adapting interventions for various populations or settings. Organizational barriers include leadership and/or organizational culture supportive of EBDM, and access to resources for EBDM (12–14). Health departments in rural areas often have less access to resources for EBDM (15). Health departments in all locations also experience high staff turnover (16–18), and few employees (less than one quarter) enter the workforce with formal training in public health (17, 19) leading to the need for periodic training to build and maintain a skilled workforce. In addition, system-level barriers can include funding, external political influence, and competing priorities. Organizational and system-level barriers may be difficult to address in the short term, though individual skill deficits can be addressed through training relatively quickly (<1 year) (7, 20, 21). Thus, training is a suitable first step agencies can take to support EBDM and build capacity (22). Understanding effective training methods for public health professionals within the context of unique barriers is important. Higher education institutions, with expertise in formally preparing the public health workforce, and who are equipped with training technology resources, can provide an advantageous training partnership for health departments.

**Figure 1:**
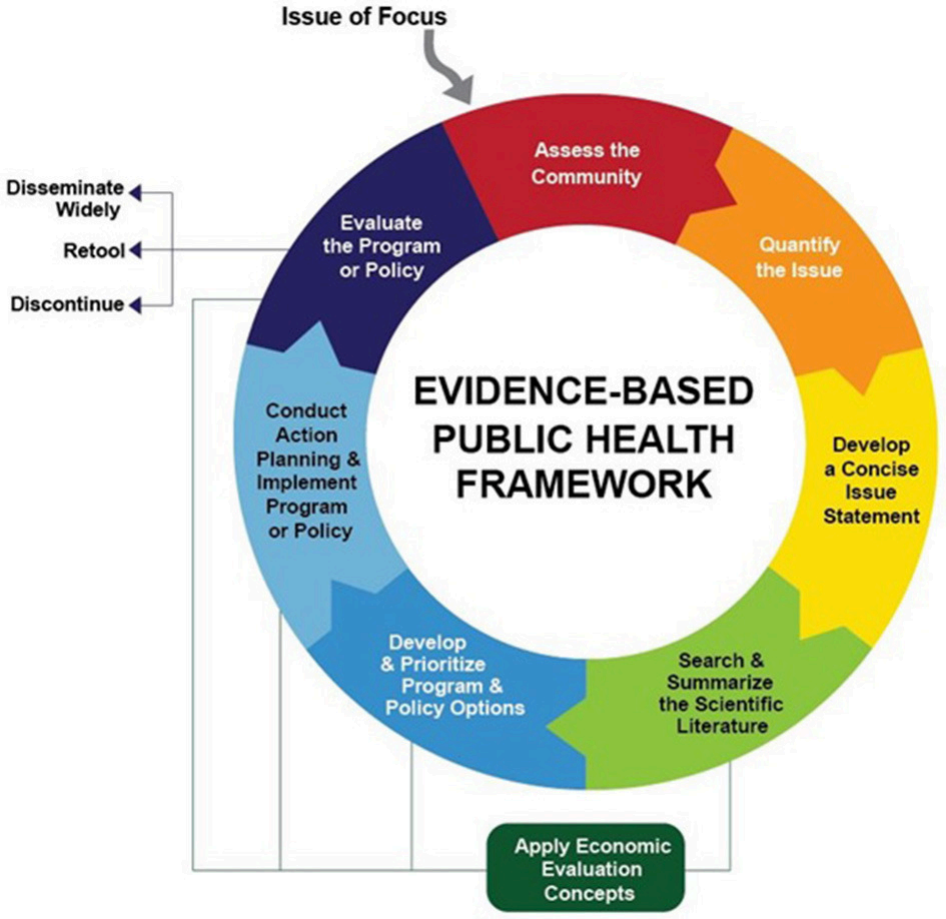
Framework for training public health professionals in evidence-based decision making.

For nearly two decades, the Prevention Research Center in St. Louis has offered Evidence-based Public Health training to public health professionals nationally and internationally on the EBDM process (Figure 1) (13, 23). The standard course provided by researchers is an in-person, 3.5 days mix of didactic and small group learning, and is effective at increasing individual skills for EBDM (20, 21, 23–25). With staff turnover, training demands are ongoing and sustainability of training programs is needed (16–18, 26). To address this, train-the-trainer models for the course were developed and utilized to scale-up trainings and reduce EBDM skill gaps (20, 27, 28). Evaluations of train-the-trainer models thus far have focused on fidelity to the original in-person delivery format, and participants have noted advantages such as small group work, use of local examples, networking with other colleagues, and in-person interaction with trainers (27, 29). Offering the course in other formats (e.g., distance learning or some combination of in-person and online) could potentially reach larger audiences such as staff in rural or satellite locations and international audiences. Distance learning methods can provide cost savings, offer a better fit for employee schedules, and/or prove more sustainable for agencies to offer regularly to staff (30–32). Scaling up capacity building approaches is a significant issue in implementation science (33). Much of the literature on public health workforce training in distance or blended learning (combination of in-person and distance) formats focuses on single topics or specific skills (31, 32, 34–43), with few focusing on the utility of training modes for scale-up potential (44, 45).

The Public Health Foundation's TRAIN Learning Network, which currently includes 25 state health departments and/or associated health divisions, provides a national web-based platform for the public health workforce to access, develop, and share trainings in various topics (46). In addition, learning networks and centers charged with educating the US public health workforce, such as Public Health Training Centers, Regional Public Health Training Centers, and the Public Health Learning Network provide distance and blended training options (47, 48). With the growing availability of web-based platforms within national and regional learning networks for public health professionals (46, 49) and increasing interest in making trainings continuously available with reduced costs, further exploring multiple modes of trainings for EBDM is necessary and timely.

The purpose of this study was to evaluate the effectiveness of distance and blended training delivery methods to reduce skill gaps in making evidence-based decisions, as compared to in-person training, as a potential option for public health professionals.

## Materials and methods

This study used a quasi-experimental design (pre-post with comparison group) to examine the effect of EBDM training on gaps in EBDM skills. The following describes the training sites, the comparison or control group, data collection and analysis, and the EBDM training modules and learning objectives.

### Training site selection and study overview

In 2014, we identified training sites that had previously requested training in EBDM and had existing collaborations across academic and health department settings. After an initial pool of 12 was identified, research staff contacted each site to determine the level of interest in collaborating as well as experience with web-based approaches for training. In total, the purposeful sample included nine training sites within Missouri, Washington, Colorado, North Carolina, Nebraska, Oklahoma, Tennessee, Vermont, and New York. Washington and Colorado included participants from outside of their state, though they were considered participants of the Washington or Colorado sites' course. The nine sites were led by five state health departments, one in-state Public Health Training Center, two Regional Public Health Training Centers, and one school of public health.

Invited site representatives included chronic disease and health promotion directors, workforce development staff, school of public health faculty, and training center staff. Site representatives agreed to: (1) recruit in-state staff and faculty to attend a train-the-trainer course in EBDM provided by the study team; (2) replicate the course in their own states or regions; and (3) support data collection for evaluation. Sites agreed to include both public health practitioners and academicians as course instructors. The study team provided technical assistance and financial support for course replication. While sites replicated the course at varying intervals in 2015–2018, and planned to continue beyond the timeframe of the funded project, this study examines pre and post-evaluations for the initial course replication at each site.

We recruited control participants through snowball sampling in health departments. We contacted directors from local health departments that were similar to participating departments based on jurisdiction population size and service to urban or rural communities. Directors identified department staff to participate in pre-post survey data collection as controls. In addition, representatives from the nine training sites identified possible contacts for controls. Control participants did not take part in the EBDM training.

### Training description

The evidence-based public health course, as originally designed and evaluated (23, 25, 27), is a 3.5 days in-person interactive training provided by university-based researchers that includes a mix of both didactic lectures and small group exercises. The course is organized into nine modules with accompanying learning objectives (Table 1) centered on the EBDM process. The nine modules cover foundational-level public health skills needed to make evidence-based decisions such as assessing needs in the community and using quantitative methods to quantify a public health issue. For example, in module three, “Quantifying the Issue,” participants complete a hands-on exercise in finding online data available for their state/county/city and become familiar with querying databases with terms for various disease rates, indicators, and outcomes. Each state has different public health governance and thus, for each course, exercises are tailored to reflect locally relevant circumstances and data and/or public health issues. While the content for exercises and/or examples varied based on relevance to states' and attendees' local health department jurisdictions, main components and learning objectives in the EBDM training are cross-cutting (Table 1) and apply to all health departments. Training site representatives attended the train-the-trainer course (27) and, along with support from study staff, planned a replication course in EBDM within their networks and recruited additional trainers from health departments and universities in their states. As courses and plans for replication developed, the core study team provided technical assistance to each site—this varied from site to site and included input on updating modules, tips on choosing course trainers, ideas for local examples, and logistics for organizing the courses. Six of the nine total sites used the in-person format and three delivered their course in a distance or blended format that combined distance and in-person components.

**Table 1:** Nine Modules of the Evidence-Based Public Health Course and Accompanying Learning Objectives.

**Module title**	**Learning objectives**
Introduction to evidence-based decision making	Understand the basic concepts of evidence-based decision making Introduce some sources and types of evidence Describe several applications within public health practice that are based on strong evidence and several that are based on weak evidence Define some barriers to evidence-based decision making in public health settings
Assessing the community	Understand the importance of conducting a community assessment, including the role of coalitions/partnerships Understand the types of data that are appropriate for assessing the needs and assets of the population/community of interest Understand the major steps in the community assessment process
Quantifying the issue	Measure and characterize disease frequency in defined populations using principles of descriptive epidemiology and surveillance Find and use various public health data sources for evidence-based decision making
Developing a concise statement of the issue	Understand the criteria for the components of a sound problem statement Develop a concise written statement of the public health problem, issue or policy under consideration in a measurable manner
Searching and summarizing the scientific literature	Understand the different types of reviews Understand the process used in systematic reviews and become familiar the Community Guide Become familiar with other web resources for public health systematic reviews Develop skills for efficient and effective literature searches and assessment Use recommended guidelines for searching the scientific literature
Prioritizing program and policy options	Identify methods for prioritizing program and policy options (Types 1, 2, and 3) Explore the role of creativity and group processes in developing intervention options. Introduce the role of group processes in adaptation of interventions
Economic evaluation	Explain the differences between types of economic evaluations most often used in public health Define key terms used in economic evaluations Describe the steps involved in conducting an economic evaluation
Developing action plans and logic models	Identify key characteristics and principles in successful action planning Understand when and how to adapt interventions for different communities, cultures, and settings Identify the steps in action planning Understand the purpose and use of logic models Be able to construct a logic model worksheet
Evaluating the program or policy	Understand the basic components of program evaluation Understand the various types of evaluation designs useful in program evaluation Understand the concepts of measurement validity and reliability Understand the contributions of both qualitative and quantitative data to the evidence based process Understand some of the methods used in qualitative evaluation Understand how to report and disseminate results Understand organizational issues in evaluation

### In-person training

Six sites provided in-person courses following the format of the original evidence-based public health course using a mix of didactic lectures, large- and small-group discussion, and small-group exercises. Most sites shortened the in-person course from the original 3.5 consecutive days to 2–2.5 consecutive days by shaving a bit of time from each module in consultation with the study team. One site delivered the in-person course in 4 days, with each day a week apart. The six in-person sites included local or in-state examples in didactic materials and exercises to make the learning relevant to current priority issues, but otherwise retained module content as provided in their train-the-trainer session.

### Distance and blended training

Three sites implemented their course either solely via online web modules, or some combination of web modules and in-person session. The first site engaged participants through nine self-paced online modules on EBDM. Each module was followed by a quiz that had to be taken in order to “pass” the module. Optional activities and reflection questions were available for participants to complete. Participants also had the option to discuss any questions through a user forum as they progressed through the course. In the second site, participants completed two online modules individually, followed by a 2-day in-person meeting and additional online modules afterwards. For the third site, training participants watched pre-recorded videos (archived online) for each module, completed homework assignments, and then attended scheduled live online learning sessions. The live distance learning sessions featured a facilitator that led module-specific activities and guided participants in further discussion.

### Survey tool

Informed by previous work (50, 51), the online survey tool comprised 51 total survey items and was used for all groups: in-person, distance or blended, and control. The survey assessed participants' agency type and university affiliation, job position, length of employment in position and in public health, gender, age, and educational degrees earned (Table 2). Participants rated each of ten skills in EBDM on their importance (1: not important to 11: very important) and “how available you feel each skill is to you when you need it either in your own skill set or among others' in your agency” (1: not available to 11: very available). We assessed the following EBDM skills:

1. Community assessment: understand how to define the health issue according to the needs and assets of the population/community of interest.
2. Quantifying the issue: understand the uses of descriptive epidemiology (e.g., concepts of person, place, and time) in quantifying a public health issue.
3. Prioritization: understand how to prioritize program and policy options.
4. Economic evaluation: understand how to use economic data in the decision making process.
5. Action planning: understand the importance of developing an action plan for how to achieve goals and objectives.
6. Adapting interventions: understand how to modify programs and policies for different communities and settings.
7. Evaluation designs: understand the various designs useful in program or policy evaluation.
8. Quantitative evaluation: understand the uses of quantitative evaluation approaches (e.g., surveillance or surveys).
9. Qualitative evaluation: understand the value of qualitative evaluation approaches (e.g., focus groups, key informant interviews) including the steps involved in conducting qualitative evaluations.
10. Communicating research to policy makers: understand the importance of communicating with policy-makers about public health issues.

**Table 2:** Baseline characteristics of survey participants by type of training method and control.

	**Overall (*n* = 272)**	**In-person only training (*n* = 84)**	**Distance or blended training (*n* = 67)**	**Control (*n* = 121)**	***P*-value^a^**
Categorical variables	*n* (%)^b^	*n* (%)	*n* (%)	*n* (%)	
**JOB POSITION**					
Top executive/health director/etc.	26 (9.6)	16 (19.0)	3 (4.5)	7 (5.8)	0.003
Administrator/deputy/Asst. Dir.	13 (4.8)	7 (8.3)	0 (0.0)	6 (5.0)	
Manager of division/program	52 (19.1)	14 (16.7)	20 (29.9)	18 (14.9)	
Program coordinator	64 (23.5)	17 (20.2)	14 (20.9)	33 (27.3)	
Technical expert (eval, epi, health edu)	46 (16.9)	9 (10.7)	14 (20.9)	23 (19.0)	
Other^c^	71 (26.1)	21 (25.0)	16 (23.9)	34 (28.1)	
**GENDER**					
Male	30 (11.0)	7 (8.3)	9 (13.4)	14 (11.6)	0.591
Female	242 (89.0)	77 (91.7)	58 (86.6)	107 (88.4)	
**AGE**					
20–29	41 (15.1)	13 (15.5)	6 (9.0)	22 (18.3)	0.016
30–39	69 (25.5)	13 (15.5)	16 (23.9)	40 (33.3	
40–49	63 (23.2)	22 (26.2)	22 (32.8)	19 (15.8)	
50–59	64 (23.6)	27 (32.1)	13 (19.4)	24 (20.0)	
60 or older	34 (12.5)	9 (10.7)	10 (14.9)	15 (12.5)	
**DEGREE**					
Master's degree or higher in any field	141 (52.2)	41 (48.8)	36 (46.3)	64 (53.8)	0.752
Public health master's or doctorate	70 (25.9)	18 (21.4)	19 (28.4)	33 (27.7)	0.524
Nursing	65 (24.1)	22 (26.2)	18 (26.9)	25 (21.0)	0.576
**AGENCY TYPE**					
Local health department	171 (65.3)	52 (61.9)	32 (51.6)	87 (75.0)	0.000
State health department	55 (21.0)	26 (31.0)	18 (29.0)	11 (9.5)	
Other agency type^d^	36 (13.7)	6 (7.1)	12 (19.4)	6 (15.5)	
Continuous variables	Mean (SD)	Mean (SD)	Mean (SD)	Mean (SD)	
Years in job position	5.01 (6.03)	5.10 (6.83)	4.99 (3.50)	4.96 (5.82)	0.986
Years in public health	10.6 (8.89)	10.91 (8.68)	10.65 (9.06)	10.34 (8.96)	0.901

a *P-value represents significance values from Pearson Chi-square test for categorical variables and one-way ANOVA tests for continuous variables across the three participant groups*.

b *Percentages are reported for valid, non-missing cases*.

c *Examples of other position include public health nurse, consultant, and faculty*.

d *Examples of other agency type include community based organization, healthcare facility, and university*.

*SD, standard deviation*.

Skills for EBDM are established as a reliable measure of effect for the in-person course in EBDM (21, 51, 52), and thus are used in this study to assess the relative effectiveness of distance or blended formats. The differences between importance and availability scores were calculated to represent gaps in EBDM skills and are the main dependent or outcome variable in this study.

### Data collection

Staggered pre-post data collection based on training date was completed between December 2014 and May 2017 with in-person, distance or blended training groups, and controls. Email invitations with a link to participate in the baseline survey (administered via Qualtrics online software) were sent 1 month or less prior to each site's training. Follow up surveys were sent 6 months post-training to all who completed the baseline survey, except in one training site where follow up surveys were not administered. Also, participants who moved to another organization or retired after the baseline survey were not invited to complete the follow up as they were deemed ineligible. Survey invitees (*n* = 857 invited at baseline and *n* = 545 invited at follow up) received both reminder emails and phone calls in order to increase response rates. The Washington University in St. Louis Institutional Review Board granted human subject exempt approval.

### Data analysis

We sought to examine the relative effectiveness of distance and blended training approaches compared to in-person courses in reducing skill gaps; thus only paired responses from individuals that completed both surveys were used for analysis in this study. Because of similarities in distance learning approaches and small numbers, the distance learning and blended trainings were combined into one group. A previous meta-analysis of internet training also assigned trainings with any type of distance learning into a single group for analysis (53) and a recent review of distance learning for professional development of rural health professionals included studies that compared blended modalities to entirely face-to-face trainings (54), lending support for the group assignment in the present study. We used descriptive statistics to examine participant characteristics and mean skill gaps at pre and post-surveys across the three participant groups: in-person only training, distance or blended training, and control. Mixed effects linear models in IBM SPSS 25 MIXED were used to assess the effect of in-person training and distance or blended training on EBDM skill gaps at post-survey compared to controls (55). Smaller gaps at post-survey indicate reduced skill gaps and/or increased skills. Adjusted models accounted for baseline EBDM skill gap, job position, years in job position, gender, age group, master's degree earned, and public health agency type based on previous research (20, 21, 31) and/or their statistically significant between-group difference at baseline between the control and training groups. Participants' state was included as a random effect to account for any between-state variation. Data analysis was completed between September and December 2017.

## Results

Response to the baseline survey was 63.9% (454 invited; 290 completed) for control and 81.6% (403 invited; 329 completed) for training groups. For the follow-up survey, the response rates for control and training groups were 48.8% (287 invited; 137 completed) and 62.0% (258 invited; 160 completed), respectively. In total, 272 post-surveys matched baseline surveys (pairs) and were used in all analyses.

Overall, most (89.0%) respondents were female, nearly two-thirds (65.3%) were from local health departments, and more than half (52.2%) held a graduate degree (Table 2). The largest (26.1%) category of selected job position was “Other,” which included a wide range of types of occupations, such as public health nurse, consultant, faculty, and planner. Between-group differences at baseline were found in job position (*p* = 0.003), age (*p* = 0.016), and agency type (*p* < 0.001) and were adjusted in multivariate modeling.

At baseline, EBDM skill gaps differed significantly across the three participant groups in two skills: community assessment (*p* = 0.018) and adapting interventions (*p* = 0.023; Table 3). Economic evaluation was the largest EBDM skill gap across all three groups at baseline and remained among the largest at follow up. Overall skill gap estimates for both in-person and distance/blended training were less at post-survey as compared to the control group (Table 4). The total mean of 10 EBDM skill gaps was significantly reduced for participants in the in-person (β = −0.55, SE = 0.27, *p* = 0.041) and distance/blended trainings (β = −0.64, SE = 0.29, *p* = 0.026) compared to controls. The gap in economic evaluation skills was also significantly reduced for both in-person (β = −0.86, SE = 0.41, *p* = 0.037) and distance/blended (β = −1.23, SE = 0.44, *p* = 0.005) training groups. Two skills, prioritization and adapting interventions, were significantly reduced (β = −0.90, SE = 0.32, *p* = 0.005 and β = −0.87, SE = 0.35, *p* = 0.013, respectively) in the in-person training group but not in the distance/blended training groups. In addition, skill gaps were reduced in quantitative evaluation (β = −0.78, SE = 0.38, *p* = 0.04) and in action planning (β = −0.66, SE = 0.32, *p* = 0.039) in the distance/blended group but not in the in-person group.

**Table 3:** Evidence-based decision making skill gaps pre vs. post-training by training delivery method.

	**In-person only training (*****n*** = **84)**		**Distance or blended training (*****n*** = **67)**		**Control (*****n*** = **121)**		***P*-value^a^**
	**Pre-mean** **(95% CI)**	**Post-mean** **(95% CI)**	**Pre-mean** **(95% CI)**	**Post-mean** **(95% CI)**	**Pre-mean** **(95% CI)**	**Post-mean** **(95% CI)**	
**EBDM SKILL GAPS**^b^							
Community assessment gap	1.77 (1.28–2.13)	**1.27**^c^ (0.86–1.59)	2.53 (1.91–3.19)	1.83 (1.25–2.39)	1.98 (1.49–2.36)	1.78 (1.31–2.22)	0.018
Quantifying the issue gap	2.37 (1.75–2.76)	2.01 (1.50–2.40)	2.37 (1.79–3.07)	1.95 (1.35–2.50)	1.97 (1.53–2.41)	1.87 (1.38–2.25)	0.803
Prioritization gap	1.99 (1.47–2.25)	**1.46** (1.07–1.85)	2.83 (2.30–3.45)	**2.03** (1.52–2.51)	2.13 (1.68–2.54)	2.41 (1.86–2.78)	0.392
Economic evaluation gap	2.97 (2.30–3.41)	2.77 (2.17–3.29)	3.55 (2.89–4.28)	2.86 (2.04–3.44)	3.04 (2.46–3.53)	3.50 (2.90–4.05)	0.269
Action planning gap	1.60 (1.17–1.99)	1.27 (0.90–1.64)	2.30 (1.74–2.91)	**1.53** (1.00–1.93)	1.83 (1.39–2.21)	1.89 (1.40–2.22)	0.139
Adapting Interventions gap	2.79 (2.24–3.25)	**2.10** (1.58–2.52)	2.68 (2.21–3.27)	2.86 (2.27–3.42)	2.57 (2.13–2.99)	2.76 (2.22–3.15)	0.023
Evaluation designs gap	2.62 (2.00–3.07)	2.15 (1.58–2.64)	3.09 (2.54–3.86)	2.58 (1.93–3.22)	2.57 (2.06–3.05)	2.61 (2.00–3.07)	0.245
Quantitative evaluation gap	1.97 (1.45–2.39)	1.71 (1.23–2.16)	2.42 (1.73–3.04)	**1.55** (1.02–2.04)	1.67 (1.16–2.09)	1.92 (1.36–2.32)	0.118
Qualitative evaluation gap	2.22 (1.61–2.67)	2.03 (1.51–2.44)	2.77 (2.20–3.33)	2.03 (1.30–2.61)	1.72 (1.17–2.16)	2.20 (1.61–2.65)	0.077
Communicating research gap	2.39 (1.76–2.83)	2.57 (1.99–3.07)	2.95 (2.34–3.62)	2.73 (2.02–3.28)	2.87 (2.38–3.32)	3.00 (2.41–3.45)	0.194
Mean of 10 EBDM skill gaps	2.27 (1.86–2.52)	1.93 (1.57–2.23)	2.75 (2.31–3.26)	**2.18** (1.71–2.60)	2.23 (1.87–2.53)	2.41 (1.95–2.71)	0.069

a *P-value column represents between group differences for pre-mean across the three participant groups*.

b *Participants were asked to rate both the importance and availability on an 11 point Likert scale (1 = not important/available; 11 = very important/available). Gaps were calculated by subtracting the Likert score rating for availability from rated importance*.

c *Bold text indicates significant difference between pre and post-mean EBDM gap scores for each group according to paired t-tests where p < 0.05*.

*EBDM, evidence-based decision making; CI, confidence interval*.

**Table 4:** Intervention effects for evidence-based decision making skill gaps by training delivery method.

	**In-person only training (*****n*** = **84)**				**Distance or blended training (*****n*** = **67)**			
	**Unadjusted^a^**		**Adjusted^b^**		**Unadjusted**		**Adjusted**	
	**β**	**SE**	**β**	**SE**	**β**	**SE**	**β**	**SE**
**EBDM SKILL GAPS**^c^								
Community assessment gap	−0.45	0.32	−0.38	0.33	−0.09	0.34	−0.20	0.36
Quantifying the issue gap	0.06	0.33	−0.06	0.34	−0.00	0.35	−0.22	0.37
Prioritization gap	−0.91^**^^d^	0.31	−0.90^**^	0.32	−0.57	0.33	−0.50	0.34
Economic evaluation gap	−0.72	0.40	−0.86^*^	0.41	−0.81	0.42	−1.23^**^	0.44
Action planning gap	−0.55	0.29	−0.57	0.30	−0.49	0.30	−0.66^*^	0.32
Adapting interventions gap	−0.74^*^	0.33	−0.87^*^	0.35	0.06	0.35	−0.24	0.37
Evaluation designs gap	−0.48	0.37	−0.68	0.41	−0.24	0.39	−0.27	0.41
Quantitative evaluation gap	−0.28	0.34	−0.29	0.35	−0.56	0.36	−0.78^*^	0.38
Qualitative evaluation gap	−0.38	0.35	−0.38	0.40	−0.60	0.38	−0.68	0.40
Communicating research gap	−0.29	0.38	−0.28	0.40	−0.29	0.40	−0.49	0.42
Mean of 10 EBDM skill gaps	−0.49	0.26	−0.55^*^	0.27	−0.44	0.27	−0.64^*^	0.29

a *Linear regression models estimating post-gap score effects (control as referent) and pre-gap score as covariate*.

b Linear regression models estimating post-gap score effects (control group as referent) adjusting for job position, gender, age category, years in public health, agency type, master degree and state as random effect.

c *Participants were asked to rate both the importance and availability on an 11 point Likert scale (1 = not important/available; 11 = very important/available). Gaps were calculated by subtracting the Likert score rating for availability from rated importance*.

d Bold text represent significantly lower skill gaps in post-survey from pre-survey compared to the control group, where asterisks represent

* *p < 0.05*,

** *p < 0.01*.

*EBDM, evidence-based decision making; β, beta value; SE, standard error*.

## Discussion

Overall, our findings are in line with previous studies (20, 21) in that training in evidence-based public health can reduce EBDM skill gaps. In this study, we specifically sought to test the utility of distance and blended training models featuring web-based learning components. This fills a significant gap in the literature. In a recent review of 20 training programs for EBDM, only three used a distance learning approach (7). We found that, like in-person training, distance and blended training models significantly reduced overall skill gaps in EBDM. The results also support continuation of the study team's train-the-trainer approach, since surveyed course participants were instructed by state-level trainers who first attended a train-the-trainer course. Below, we discuss the implications of distance and blended trainings for public health professionals as a possible supplement to in-person approaches to building capacity for EBDM as well as next steps to further apply the learnings.

### Comparing delivery methods

We found that training via in-person or distance and blended approaches had similar effects in reducing overall gaps in EBDM skills. Similarly, Sitzmann and colleagues' meta-analysis of the comparative effectiveness of web-based and classroom-style trainings show that overall, effects tend to be similar across the trainings (44). While we did not examine each variety of distance or blended trainings separately due to small subgroup numbers, Sitzmann et al.'s findings suggest a potentially larger effect for combination trainings that integrate both web-based formats and in-person components. Sitzmann et al. caution training developers against completely replacing in-person instruction with web-based modalities. Instead, enhancing existing components with either in-person or web-based counterparts could be alternatively effective, as well as offering a choice of delivery methods. For example, the meta-analysis showed larger effects in studies where learners self-selected into web-based trainings when compared to experimental designs. Thus, autonomy may be key. Our study did not inherently offer a choice to each individual, but sites themselves were autonomous in creating the delivery method that would be most advantageous for their staff and partners. Allowing sites to select their training delivery method may have contributed to the overall success of the training in reducing EBDM skill gaps. Limitations that Sitzmann et al. pose as possible next steps for examination are level of interaction with learners, learners' experience with computers or other web-based trainings, and course quality, as these may also contribute to effectiveness of delivery method.

### Offer diversity in training delivery methods

The public health workforce come from many professional backgrounds, and less than one quarter have formal training in public health (17, 19). Training in EBDM is thus a common need across the workforce spectrum. With funding traditionally attached to specific programs (e.g., grant awards for diabetes and colorectal cancer screening programs), public health has become a workforce with diverse but program specific skills. The EBDM process cuts across silos of public health funding to examine foundational principals such as involving stakeholders in a community assessment, designing evaluations before programs are underway that capture the full impact of services, and effectively communicating the value of programs to policy makers who can sustain or allocate funding. This process is integral to public health practice and is why increasing skills in EBDM among the workforce has the potential to affect population health. If training is a key ingredient to enhancing EBDM skills, any training approach should consider the end user. Our study suggests that distance and/or blended learning for public health professionals can reduce skill gaps in EBDM similarly to in-person training. Kaufman and colleagues conducted key informant interviews with public health agency employees regarding sustainable change through trainings at health departments; a common theme that arose for producing sustainable change was to offer combinations of approaches (in-person training, on-site learning, distance learning, peer learning collaboratives, etc.) (56). Training modalities are not one-size-fits-all. Formative research with groups before development and implementation should be used to determine what is needed by the training's target audience (30, 34). Unique individuals make up the public health workforce, and we should be cognizant of the diverse needs of those individuals. This includes assessing the advantages and/or disadvantages of training modalities for the various public health settings in which professionals operate.

### Train-the-trainer model

Regardless of delivery style, the instructors for the EBDM courses evaluated in this study were state-level trainers who attended a train-the-trainer course provided by the study team. Benefits of the EBDM course as delivered by the study team have been documented previously (23, 25). Similar benefits were found in four sites with courses led by state-level instructors following training by the study team (20, 27). This study's assessment was more detailed and shows the EBDM courses reduced specific participant skill gaps when taught by state-level trainers who were previously taught by the core trainers in St. Louis. Reduced skill gaps were found both among attendees of in-person courses and participants in distant and/or blended courses. This has positive implications for scale-up of ongoing EBDM training delivered via in-person, distance, or blended formats. Partly because of staff turnover in public health agencies (16, 18), ongoing periodic EBDM trainings will continue to be needed.

### Training delivery considerations

EBDM is not a process carried out by a sole worker. EBDM requires collaboration with colleagues, stakeholders, and community members. Therefore, learning the basics of EBDM among peers, in-person, has many advantages, such as the ability for small group work—learning from each other, having one-on-one questions answered by trainers in the room, and networking with other colleagues—all of which have been cited as facilitators of learning in in-person courses (23, 27). However, online training platforms are increasingly able to integrate more interactive features which allow for similar experiences. In addition, web-based training has the potential to reach parts of the public health workforce that may not otherwise have access to in-person training (e.g., rural workforce, not in close proximity to schools of public health/trainings). Web-based trainings also provide the flexibility for those whose schedules are too demanding for a 3.5 days intensive in-person training, but could learn at their own pace, though immersion offers a chance to focus solely on training without the distraction of day-to-day work activities. Finally, purely web-based models have the potential to reach larger audiences and can perhaps do so more quickly if they are offered frequently or on demand. In-person trainings traditionally accommodate about 35 individuals. One site in our study, which offered their web-based training without any in-person component, trained well over three times that. Web-based trainings offer the opportunity to quickly and simultaneously push consistent and standardized messaging around a topic or skill to all employees.

Costs to participants can be reduced without full day trainings (that often require travel, food, etc.), though upfront costs to an organization to develop and launch web-based training can be high and money should be budgeted for ongoing updates to trainings. One way organizations can minimize their costs (which some sites did in this study) is to integrate web-based modules or training components into already existing web-training platforms. One example is TRAIN, a national learning collaborative that allows web-access options to develop and disseminate professional development trainings with an organizational subscription. Currently, 25 state health departments are affiliated TRAIN sites, and subscriptions for local public health agencies are also available (46). Regional and in-state Public Health Training Centers can also potentially offer needed resources for training infrastructure that could reduce upfront costs to organizations. To support sustainability of any ongoing training programs, regardless of format, formal plans to acquire and designate resources are needed (39).

### Next steps

This is an exciting time to be in public health. Much focus is placed on creating and maintaining a competent workforce capable and passionate about keeping populations of people healthy. Learnings from this study can infuse current plans to spread capacity for EBDM through training efforts, and more importantly, can inform further, more rigorous studies to explore with more depth the implementation variations in blended or distance course delivery.

### Limitations

While our study provides important findings regarding the use of alternative approaches to training public health professionals in-person, findings should be interpreted within the context of several limitations. All data presented are self-reported, which introduces the potential for social desirability bias. This study did not ascertain behavior change (level of using EBDM processes before and after training), an important piece of evaluating training outcomes (57). Three participant characteristics differed across training and control groups, but did not drive the reported adjusted findings per additional analyses conducted. Sites also differed in their implementation of the training, and detailed implementation differences were not directly measured. Because of small numbers, the distance learning and blended trainings were combined, thus we were not able to fully isolate differences between full distance learning and either partial or full in-person training. Future studies are needed to determine the level of effect by particular distance and/or blended model.

## Conclusions

The public health workforce is a diverse group of professionals. The spread of EBDM within the workforce has the potential to decrease disease burden in the population. Building capacity for EBDM through training is not a one size-fits-all approach—offering multiple training modes increases the potential for scaling up and sustaining EBDM across the public health workforce.

## Availability of data and material

Raw data will be made available by the authors, without undue reservation, to any qualified researcher upon request.

## Author contributions

RB and PA conceptualization and design, RB, PA, PE, and KD survey instrument development, KA and SY data collection, RJ data management and data analyses. All authors read and approved the final manuscript.

### Conflict of interest statement

RB is currently a guest associate editor for Frontiers in Public Health, Public Health Education and Promotion. KD is employed by Focus Pointe Global. The remaining authors declare that the research was conducted in the absence of any commercial or financial relationships that could be construed as a potential conflict of interest.

## Abbreviations

EBDM: evidence-based decision making
